# Survival risk prediction in hematopoietic stem cell transplantation for multiple myeloma

**DOI:** 10.1515/jib-2024-0053

**Published:** 2025-06-03

**Authors:** Jose María Belmonte, Miguel Blanquer, Gregorio Bernabé, Fernando Jiménez, José Manuel García

**Affiliations:** Computer Engineering Department, Faculty of Computer Science, 16751 University of Murcia, 30100, Murcia, Spain; Hematology Department, Hospital Virgen de la Arrixaca and IMIB, 30120, Murcia, Spain; Department of Information and Communication Engineering, Faculty of Computer Science, University of Murcia, 30100, Murcia, Spain

**Keywords:** artificial intelligence, feature importance, machine learning, survival analysis, survival predictions

## Abstract

This paper investigates the application of *Survival Analysis* (SA) techniques to forecast outcomes after *autologous Hematopoietic Stem Cell Transplantation* (aHSCT) for *Multiple Myeloma* (MM). By leveraging six SA models, we examine their predictive capabilities, measured through the *Concordance Index* (C-index) metric. Beyond evaluating model performance, we analyze feature importance using permutation and SHAP methods, highlighting key clinical factors such as treatment history, disease stage, and prior disease progression or relapse as critical predictors of survival. The findings suggest that while all models performed well based on the C-index, a detailed examination revealed variations in how each model processed data. Specifically, the Coxnet and Random Survival Forest models exhibited a more thorough use of clinical variables, whereas the gradient boosting models appeared to rely on a narrower range of features, potentially limiting their ability to differentiate between patients with comparable profiles. Risk predictions categorized patients into low, moderate, and high-risk levels. For lower-risk patients, the procedure showed positive outcomes, while higher-risk individuals were predicted to have limited survival benefits, recommending alternative treatments. Lastly, we propose future research to expand these models into time-to-event estimations, offering additional support for decision-making by predicting patient life expectancy post-transplant, considering their pre-transplant clinical attributes.

## 1 Introduction

*Autologous Hematopoietic Stem Cell Transplantation* (aHSCT) represents a common and effective therapeutic option for treating hematologic cancers, particularly in cases of leukemia and *Multiple Myeloma* (MM) [1], 2]. However, predicting individual outcomes remains a significant challenge, emphasizing the role of advanced predictive models. This procedure involves harvesting and reintroducing the patient’s own stem cells to help recover bone marrow function after intensive chemotherapy [3]. While aHSCT has been proven to significantly extend patient survival and improve their quality of life, predicting individual outcomes, such as *Overall Survival* (OS), remains essential in order to customize treatment plans and provide patients with accurate prognostic information.

The incorporation of *Artificial Intelligence* (AI) and *Machine Learning* (ML) into healthcare systems has transformed multiple medical fields by introducing advanced tools for diagnosis, prognosis, and optimizing treatments [4]. These technologies empower clinicians with sophisticated techniques for analyzing complex clinical datasets, which in turn support the development of predictive models that enhance decision-making and improve patient care. In recent years, AI and ML have gained significant traction in areas such as oncology and transplantation, where variability in patient outcomes poses substantial challenges for accurate prediction.

Censored data is a recurrent issue in medical research, particularly when some patients do not experience the event of interest (e.g., death or disease relapse) within the study period. This challenge is especially pronounced in areas like transplantation, where varying follow-up periods and many patients remaining event-free complicate the analysis [5]. Traditional ML methods – such as scoring systems and standard ML classifiers – often fall short in handling time-to-event data, especially in the presence of censored observations, where the event of interest (e.g., death or relapse) has not occurred during the study period or remains unknown. Additionally, many of these models focus on binary or continuous outcomes, often overlooking the time component that is critical in clinical scenarios like HSCT. This can result in biased survival estimates and reduce the model’s ability to capture patient-specific risk trajectories over time. Furthermore, traditional approaches generally fail to incorporate censored observations effectively, leading to incomplete or inaccurate risk stratifications.

To overcome these limitations, *Survival Analysis* (SA) emerges as a robust method for analyzing time-to-event data, offering more accurate and interpretable predictions. One of the notable advantages of SA models is their ability to estimate the probability of an event occurring over a specified time period. In the context of aHSCT, these models are employed to predict which patients are more likely to face adverse outcomes following the procedure or based on pre-transplant clinical factors.

While AI and ML models offer powerful tools for risk prediction and clinical decision-making, their application in high-stakes medical scenarios such as HSCT raises important ethical considerations. The potential impact of predictive models on treatment decisions underscores the need for transparency, interpretability, and fairness. Clinicians must be able to understand how and why a model arrives at a particular prediction, especially when these predictions influence critical decisions regarding patient care. Additionally, ensuring that models are free from biases – whether arising from historical data or model design – is crucial to maintaining equity in healthcare delivery. Data privacy and the responsible handling of sensitive patient information further reinforce the importance of ethical oversight when integrating AI/ML tools into clinical workflows.

In this study, we build upon the methodology proposed in our previous research [6] in SA for aHSCT, refining the approach with an updated dataset with enhanced preprocessing and improved hyperparameter tuning for the SA models. These refinements significantly improved the Concordance Index (C-index) of over 0.82 across all models. Additionally, we performed a more exhaustive feature importance analysis, now applied to the fully encoded dataset. This analysis exhibits that while all models show strong predictive capabilities, Coxnet and Random Survival Forest stood out by incorporating a wider range of attributes, better differentiating between patients with similar characteristics, and aligning with medical literature and expert opinion. The most significant attributes identified for predicting post-transplant outcomes were the International Staging System (ISS), disease relapse or progression, pre-transplant chemotherapy, and disease status. We further validate these models on four new patients who underwent transplantation in the last four years. We stratified the predicted risks based on their pre-transplant characteristics and aligned these results with survival outcomes. This risk stratification enables clinicians to assess whether a transplant genuinely benefits the patient or if alternative treatments should be considered.

Our primary objective is to help clinicians determine whether the potential risks of a transplant outweigh its benefits. By evaluating pre-transplant characteristics and corresponding risk profiles, this approach provides valuable insights into whether proceeding with a transplant is appropriate for a particular patient. Such risk stratification is essential for personalizing treatment plans, ensuring that patients are not subjected to unnecessary or high-risk procedures, and ultimately promoting more informed and patient care.

The rest of this paper is structured as follows: Section 2 reviews related work, Section 3 outlines the main components of the study, Section 4 presents the experiments and analysis, and Section 5 presents conclusions and future work.

## 2 Related works

The application of AI and ML in *Hematopoietic Stem Cell Transplantation* (HSCT) is becoming more widespread as these technologies are leveraged to tackle a variety of clinical issues.

Muhsen et al. [7], 8] underline the potential of AI to enhance many facets of HSCT, including improving diagnostic accuracy, refining prognostic models, and optimizing therapeutic approaches. Their work also emphasizes the role of AI in donor matching and in forecasting complications like graft-versus-host disease.

Regarding the prediction of donor availability, various ML algorithms, including boosted decision trees, have demonstrated high effectiveness, achieving remarkable accuracy in identifying compatible hematopoietic stem cell donors [9]. A comprehensive review of ML methodologies applied in HSCT highlights the frequent use of ensemble techniques, regression models, Bayesian approaches, and support vector machines, which predominantly use clinical and genetic data to drive their predictions [10].

Taheriyan et al. [11] also explored how ML models can be utilized to forecast outcomes in HSCT, emphasizing the strong performance of techniques such as Deep Learning and Bayesian Networks. Likewise, Ratul et al. [12] investigated survival predictions for pediatric HSCT patients, utilizing a dataset that shares similar attributes with ours, although they primarily employed traditional ML classifiers as opposed to SA models. Their study demonstrated the usefulness of these methods but did not address the handling of censored data, a key aspect in SA. Furthermore, Ivanics et al. [13] expanded on this topic within transplant oncology, highlighting how AI and ML are applied to refine clinical predictions and improve data analysis in the transplantation context.

SA, a statistical method designed to estimate the probability of an event occurring over time, is pivotal in clinical research, particularly when dealing with censored data. However, working with such data is often challenging due to varying follow-up periods and the presence of right-censored cases, where the event has not yet occurred [14]. In 2020, Pölsterl [15] introduced *scikit-survival*, a Python library that integrates smoothly with *scikit-learn*, providing a range of SA tools specifically for time-to-event analysis and model evaluation.

These studies collectively highlight the growing importance of AI and ML in HSCT while also pointing to the need for further research into the most appropriate methodologies, such as SA, and the identification of key data variables to enhance predictive accuracy in this field.

## 3 Materials and methods

**Figure 1:**
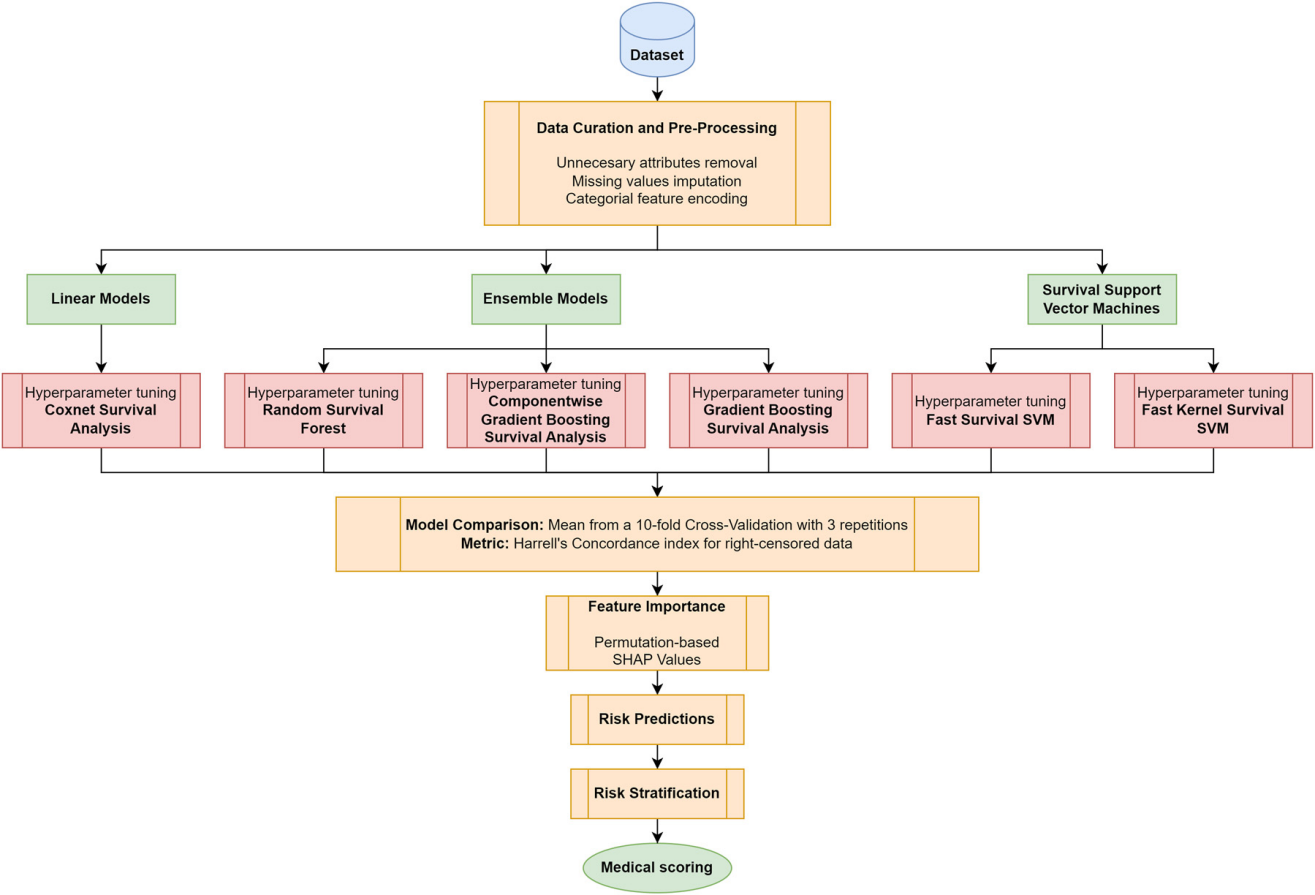
Flowchart for risk predictions outcomes.

In this section, we outline the key components of our study, beginning with a description of the dataset, including details about its curation and pre-processing steps. Following that, we provide an overview of the various SA models that are compared and assessed. This section also includes a feature importance analysis and an explanation of how survival risk predictions are conducted. A flowchart illustrating the process is provided in Figure 1.

### 3.1 Dataset description and pre-processing

The dataset utilized in this study is provided by the Hematology Department of the University Hospital Virgen de la Arrixaca in Murcia, and contains medical records of transplants performed between 1995 and 2021, with patient follow-up extending up to the latter year.

Initially, the data was compiled into a spreadsheet consisting of 1,115 records and 48 attributes to track the progress of patients as monitored by healthcare professionals. The attributes covered a range of diagnoses requiring hematopoietic stem cell transplants, including both allogeneic and autologous procedures, as well as post-transplant information.

For the purposes of this study, we filtered the dataset to include only autologous transplants in patients diagnosed with MM. After removing irrelevant attributes and crafting time-related features, the resulting dataset comprised 177 instances and 12 attributes.

As shown in Table 1, most of the attributes are categorical, except for the patient’s age at the time of transplantation. To effectively handle missing values – a common issue in clinical datasets – a multi-step imputation strategy was applied. Firstly, domain experts reviewed the dataset and manually completed missing entries when supporting clinical information was available from external sources, such as patient medical records or laboratory results. This approach ensured that critical clinical variables were imputed based on accurate and context-specific data. For cases where manual completion was not feasible, an AI-based imputation method was employed using the *k*-Nearest Neighbors algorithm.

**Table 1:** Dataset attributes description.

*N*°	Attribute	Description	Values
1.	STAGE	Cancer extension classification	IIA, IIB, IIIA, IIIB
2.	ISS	International staging system	1, 2, 3
3.	CONDITIONING	Pre-transplant chemotherapy	MELFALAN [70, 140, 200]
4.	HEMODIALYSIS	Recipient with renal impairment hemodialysis	Yes, No
5.	AGE	Recipient’s age at transplant	Numerical (19–75 years)
6.	N° HSCT	Number of previous HSCT	1, 2
7.	DISEASE STATUS	Disease status at HSCT	CR, PR, VGPR^a^
8.	DRI	Recipient’s disease risk index	Intermediate, high
9.	PREV NEOPLASIA	Previous neoplasia (different from MM)	Yes, No
10.	PROGR RELAP	Progression or relapse of the disease	Yes, No
11.	DECEASED	Survival status	Yes, No
12.	SURVIVAL TIME	Time of observation or time to death in months	Numerical (1–201 months)

^a^CR, complete response; PR, partial response; VGPR, very good partial response.

Since these models cannot directly process nominal variables, we transformed the categorical attributes into numerical format using the *encode_categorical()* function from the *sksurv* library, encoding attributes with *M* categories into *M-1* binary variables using one-hot encoding.

### 3.2 Survival models and risk predictions

The metric utilized to assess the performance of survival prediction models, known as the C-index, gauges the model’s capability to accurately rank individuals based on their predicted risks and observed survival times. Specifically, the C-index measures how effectively the model differentiates between two randomly selected patients, one of whom experiences the event of interest before the other. A pair is considered concordant if the patient with the higher predicted risk indeed experiences the event earlier. The C-index score is computed as the ratio of concordant pairs to all comparable pairs, with a score of 1.0 indicating perfect concordance (the model accurately ranks all patients), while a score of 0.5 suggests random prediction.

The selection of these SA models from the Python library *scikit-survival* [15], was driven by their well-established use in time-to-event data analysis. These models can be categorized into three main types:

– *Linear Models*, such as *Cox’s proportional hazard with elastic net penalty* (Coxnet), model the relationship between covariates (independent variables) and individual survival times. This model posits that the hazard function is a product of a baseline hazard function and an exponential function of a linear combination of covariates, without imposing assumptions on the shape of the baseline hazard, thus classifying it as semi-parametric.
– *Ensemble Models* amalgamate multiple models to enhance predictive performance and robustness, adeptly capturing intricate relationships in the data and managing non-linear patterns that might be overlooked by individual models. Notable ensemble models include *Random Survival Forest* (RSF), derived from traditional Random Forests, *Gradient Boosting* (GB), and *Componentwise Gradient Boosting* (CWGB).
– *Survival Support Vector Machines* (SVMs) seek to identify a hyperplane that optimally separates the data into distinct classes. In the context of SA, these classes correspond to different survival times, ranked using a loss function that penalizes prediction errors. SVMs are particularly effective in high-dimensional data scenarios and can capture both linear and non-linear relationships. The SVMs employed in this study are the *Fast Survival Support Vector Machine* (FS) and the *Fast Kernel Survival Support Vector Machine* (FKS).

This diverse set of models ensures a comprehensive evaluation of survival prediction techniques, balancing accuracy, interpretability, and computational efficiency.

The process for utilizing these models is as follows. First, each model undergoes optimization by identifying the best hyperparameters, which involves creating a dictionary containing the model’s parameter settings and employing the C-index metric as a means to evaluate the performance of the cross-validated model.

Subsequently, each model is assessed using a 10-fold cross-validation with three repetitions on the dataset. This approach entails dividing the dataset into multiple subsets, or folds, and systematically training and testing the model across various combinations of training and validation sets. Utilizing cross-validation enables us to evaluate the model’s generalization capabilities over different data subsets, strengthening the evaluation against the limitations posed by a single static train-test split and reducing the risk of overfitting that may occur when using the entire dataset for both training and evaluation.

The SA models utilized in this research apply the *predict()* method to generate a risk score, which is an arbitrary value indicating the likelihood of the event occurring sooner for a particular patient compared to others in the dataset. This risk score is typically unbounded and not linked to a specific scale, serving primarily as a relative indicator of risk rather than an absolute probability.

In practice, higher risk scores signify that a patient is more likely to experience the event of interest earlier, whereas lower risk scores imply a longer anticipated time until the event occurs. This prediction mechanism allows for the ranking of patients based on their predicted risk, which is especially beneficial in clinical decision-making. Clinicians can leverage these risk scores to prioritize patients for closer observation, make informed choices regarding the necessity of proceeding with a transplant, and assess the potential requirement for additional interventions.

### 3.3 Feature importance techniques

To gain insights into the significance of each variable, we employ feature importance methods, specifically *permutation importance* and *SHAP* (Shapley Additive exPlanations), within our SA models.

– *Permutation Importance.* This technique evaluates the importance of each feature by randomly shuffling its values and observing the resultant impact on the model’s performance. During this process, the values of a feature are permuted, and the model is re-evaluated using the modified data. The change in performance serves as a measure of that feature’s significance. A positive permutation importance indicates that the feature contributes positively to the model’s predictive accuracy – shuffling it degrades performance. Conversely, a value near zero suggests minimal impact. In contrast, negative values imply that the feature might introduce noise or mislead the model, as the model performs better when the feature is randomized. This can occur in cases where the feature is weakly informative or interacts negatively with other variables.
– *SHAP.* This method, grounded in game theory, allocates a contribution value to each feature for every model prediction. It calculates the contribution of each feature for each prediction by considering all possible combinations of features. Ultimately, it leverages game theory to assign a value to each feature, representing its average contribution across all scenarios. The sign of the *SHAP* values indicates the direction of the feature’s effect, depending on whether a feature increases or decreases the model’s prediction relative to the baseline; the magnitude of the SHAP value reflects the strength of the feature’s influence on the prediction.

While both *permutation importance* and *SHAP* values offer valuable insights into model interpretability, they present certain limitations, particularly in the context of clinical data. *Permutation importance* can be sensitive to correlated features, potentially underestimating the importance of variables that share predictive power or may produce unstable results in smaller datasets or when dealing with high-dimensional data. On the other hand, *SHAP* values, despite providing more granular and consistent feature attributions, assume feature independence and can be computationally intensive.

Following the methodology outlined in the previous section, we conduct a 10-fold cross-validation with three repetitions. Both feature importance techniques are applied to the resulting 30 subsets throughout this process, and the mean outcomes are calculated.

By utilizing feature importance techniques, we aim to identify which features play the most pivotal role in determining the predicted survival outcomes for each model. This analysis enables us to assess the consistency of results across various models and to ensure that the most significant attributes identified correspond with established medical literature on the key factors influencing patient prognosis following aHSCT.

In conclusion, the analysis of feature importance is essential not only for validating the predictive capabilities of the models but also for enhancing their interpretability and ensuring alignment with clinical knowledge. This exploration provides deeper insights into how effectively the models identify the most relevant factors influencing patient outcomes, ultimately facilitating more informed clinical decision-making.

## 4 Results and discussion

In this section, we present the performance outcomes of the evaluated SA models as measured by the C-index metric. Following this, we conduct a detailed analysis of attribute importance to identify the most significant features for each model, utilizing the feature importance techniques discussed previously. Lastly, we showcase the predictions’ results, outlining the outcomes for a selected group of patients and emphasizing the models’ predictive capabilities in practical scenarios. This comprehensive analysis enables us to better understand SA models’ effectiveness and predictive power in the context of aHSCT for MM.

### 4.1 Survival models: C-index comparison

Table 2 displays the mean C-index results obtained from training and evaluating the models through 10-fold cross-validation with three repetitions, along with the hyperparameters selected during the optimization process. These results reflect the average performance across the 30 individual splits for each of the six models.

**Table 2:** C-index comparison across all models.

SA models	Hyperparameters	C-index 3 rep 10-fold CV mean
**Penalized cox (coxnet)**	Default	**0.8817**
Random survival forest (RSF)	{’min_samples_leaf’: 1, ’min_samples_split’: 2}	0.8464
Componentwise gradient boosting (CWGB)	Default	0.8528
Gradient boosting (GB)	{’learning_rate’: 0.05, ’max_depth’: 1, ’subsample’: 0.5}	0.8656
Fast survival SVM (FS)	{’Alpha’: 1.52587890625e-05, ’optimizer’: ’Avltree’}	0.8221
Fast kernel survival SVM (FKS)	{’Alpha’: 0.00024414, ’kernel’: ’Linear’, ’optimizer’: ’Avltree’}	0.8397

Bold values indicate the model with the best performance according to the Concordance Index (C-index).

Overall, the C-index comparison results indicate that all SA models demonstrate a commendable level of concordance, with minimal variation observed among them. Each model consistently achieves a performance score exceeding 0.82, highlighting their efficacy in predicting patient outcomes based on OS.

### 4.2 Feature importance analysis

While all models demonstrated strong performance according to the C-index, it is crucial to conduct a comprehensive analysis of how these models interact with the attributes in our dataset.

Comparing the results of feature importance across different models, we can identify whether certain clinical parameters consistently emerge as significant predictors of risk, and whether these findings align with established research on aHSCT. In this study, the ISS, history of disease progression or relapse, and the number of previous treatment lines consistently emerged as strong predictors across multiple models, supporting their recognized roles as critical risk factors in the clinical literature. This reinforces the reliability of the models in identifying key determinants of survival outcomes. Therefore, it is vital to verify if these attributes are similarly represented in the models’ behaviors. Any inconsistent or unexpected results may warrant further investigation into potential biases or limitations within the models.

Tables 3 and 4 present the ranges of values obtained from the two feature importance techniques applied to all models.

**Table 3:** Impact of feature permutation on model performance.

Attribute	Coxnet	RSF	CWGB	GB	FS	FKS
ISS = 3	0.3066	0.2288	0.2706	0.2519	0.1536	0.1791
PROGR RELAP = Yes	0.0760	0.0327	0.0357	0.0379	0.0393	0.0355
ISS = 2	0.0303	0.0045	0.0059	0.0048	−0.0257	−0.0125
HEMODIALYSIS = Yes	0.0068	0.0011	0.0000	0.0000	0.0011	−0.0009
STAGE = IIIA	0.0020	−0.0063	0.0000	0.0000	0.0027	−0.0010
DISEASE STATUS = PR	0.0014	−0.0018	0.0000	0.0003	0.0109	0.0038
STAGE = IIA	0.0002	0.0019	0.0000	0.0000	0.0080	0.0049
STAGE = IIB	−0.0002	0.0000	0.0000	0.0000	0.0001	0.0003
STAGE = IIIB	−0.0007	−0.0008	0.0000	0.0035	0.0112	0.0057
CONDITIONING = MELFALAN70	−0.0013	0.0020	0.0000	0.0000	−0.0003	0.0008
CONDITIONING = MELFALAN140	−0.0015	0.0044	0.0000	0.0000	0.0043	−0.0014
AGE	−0.0018	0.0008	0.0000	−0.0013	0.0016	0.0039
DRI = Intermediate	−0.0024	−0.0001	0.0000	−0.0007	0.0016	−0.0008
CONDITIONING = MELFALAN200	−0.0026	−0.0009	0.0000	−0.0011	0.0016	0.0001
DISEASE STATUS = VGPR	−0.0030	−0.0014	0.0000	−0.0003	0.0014	−0.0017
Nº HSCT = 2	−0.0032	−0.0082	−0.0021	−0.0029	−0.0027	−0.0082
PREV NEOPLASM = Yes	−0.0045	−0.0041	0.0000	0.0000	−0.0000	−0.0011

**Table 4:** Feature importance based on SHAP values.

Attribute	Coxnet	RSF	CWGB	GB	FS	FKS
ISS = 3	1.5823	10.8804	0.8942	0.7502	0.0172	0.1210
PROGR RELAP = Yes	−1.2739	−2.4875	−0.0751	−0.1442	−0.0095	−0.0648
ISS = 2	−0.1011	−0.0247	−0.0076	−0.0030	0.0001	−0.0016
HEMODIALYSIS = Yes	0.0793	0.0997	–	–	−0.0002	−0.0006
STAGE = IIIA	0.0100	−0.1288	–	–	−0.0006	−0.0027
DISEASE STATUS = PR	−0.0021	−0.2761	–	−0.0000	0.0004	0.0009
STAGE = IIA	0.0123	−0.0423	–	–	0.0006	0.0019
STAGE = IIB	−0.0008	−0.0223	–	–	−0.0001	−0.0005
STAGE = IIIB	0.0067	0.1708	–	0.0014	0.0004	0.0034
CONDITIONING = MELFALAN70	−0.0357	0.1298	–	–	0.0000	0.0005
CONDITIONING = MELFALAN140	−0.0598	0.1085	–	–	−0.0008	−0.0037
AGE	−0.0110	0.2449	–	0.0000	−0.0000	−0.0008
DRI = Intermediate	0.0145	0.1965	–	0.0003	0.0001	0.0007
CONDITIONING = MELFALAN200	−0.0064	0.1243	–	0.0003	−0.0000	−0.0014
DISEASE STATUS = VGPR	−0.0383	−0.2024	–	−0.0003	0.0001	−0.0006
N**°** HSCT = 2	0.0012	0.7028	0.0006	0.0010	0.0014	0.0075
PREV NEOPLASM = Yes	0.0290	0.1078	–	–	0.0000	0.0002

The findings across all evaluated models reaffirm the critical impact of key clinical variables, particularly the ISS, disease progression or relapse, and the number of treatment lines, on survival outcomes following MM aHSCT.

However, some deviations from expected patterns were observed. For example, while age is traditionally considered a significant factor in transplant outcomes, certain models assigned it a lower relative importance compared to other clinical variables. This could suggest that age may have a more nuanced impact when considered alongside other comorbidities or treatment factors.

While models based on gradient boosting achieve strong C-index scores, they tend to focus on a limited set of key attributes, potentially neglecting clinically relevant variables. This narrow focus can reduce the model’s ability to differentiate between patients with similar characteristics, limiting its effectiveness in complex scenarios. These findings highlight that, beyond traditional performance metrics like the C-index, evaluating the interpretability and clinical relevance of feature importance is essential for robust risk stratification.

Unexpected patterns or model inconsistencies could indicate areas where data complexity, feature interactions, or sample size limitations influence the model’s behavior. Addressing these gaps through further research could enhance the accuracy and clinical applicability of predictive models in HSCT.

Consequently, although a preliminary analysis based solely on the C-index may suggest that any of these models could be appropriate for clinical application, a more thorough examination of feature importance reveals that models like Coxnet or those utilizing decision trees (e.g., RSF) offer a more balanced and comprehensive analysis of all available data, allowing for a more nuanced interpretation of patient characteristics.

### 4.3 Risk stratification

To evaluate the effectiveness of the SA models, we randomly selected four patients from the dataset and performed risk predictions following their aHSCT. The predictions can be validated since we have access to the actual status and survival times for these patients. These individuals were excluded from the training set to assess the generalization performance of our predictive model. Table 5 summarizes the key characteristics of the four selected patients.

**Table 5:** Test patients’ main characteristics.

	Stage	ISS	Conditioning	Age	N° HSCT	Status	DRI	Relapse	Survival time
P1	IA	1	MELFALAN70	46	1	CR	Intermediate	No	Alive after 47 months
P2	IIIB	2	MELFALAN140	52	2	PR	Intermediate	No	Alive after 26 months
P3	IIIA	2	MELFALAN200	64	2	VGPR	Intermediate	Yes	Deceased after 39 months
P4	IIIA	3	MELFALAN200	72	2	PR	High	Yes	Deceased after 10 months

Considering the values of their parameters and the most influential attributes for overall survival identified in the preceding section, we can anticipate a favorable prognosis for patients 1 and 2, a moderate risk for the third patient, and an unfavorable prognosis for the final patient due to a high ISS, advanced disease stage, and a history of relapse post-transplantation. Table 6 displays the predicted risk scores for each patient, with each model predicting this index on an arbitrary scale. Higher risk scores indicate a lower probability of survival following the transplant procedure.

**Table 6:** Risk score prediction.

	Coxnet	RSF	CWGB	GB	FS SVM	FKS SVM
Patient 1	−6.00	8.34	0.00	−1.13	−0.01	−0.02
Patient 2	−3.63	18.28	0.00	−1.11	0.01	0.10
Patient 3	1.14	24.12	0.28	−0.59	0.04	0.32
Patient 4	7.57	64.36	3.33	2.16	0.16	0.93

**Figure 2:**
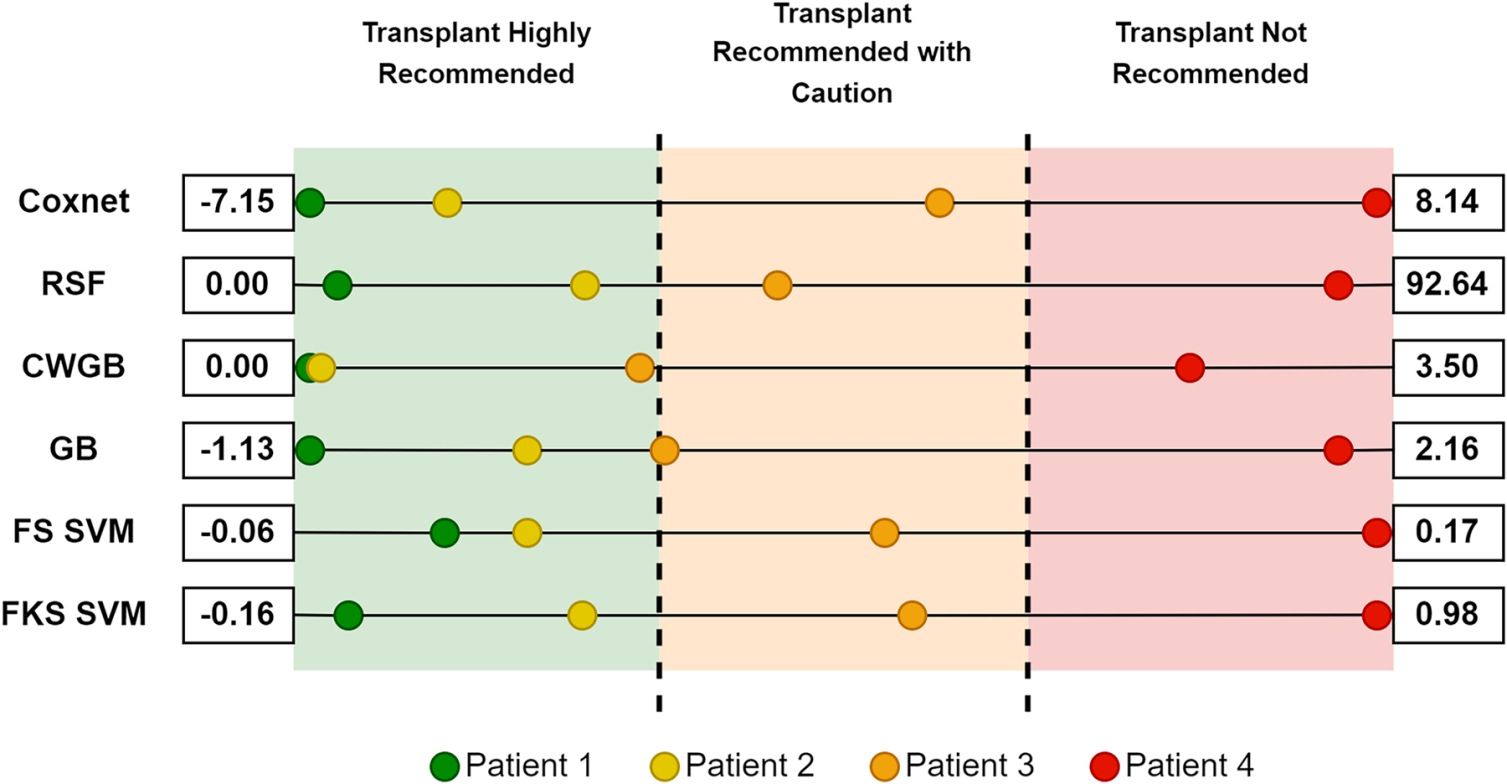
Predicted risk scores for patient 1 (green), patient 2 (yellow), patient 3 (orange) and patient 4 (red) within the range of risks for each model with the best and worst diagnosis.

While we observe that the predicted risk scores increase consistently across patients with each model, these scores can still be challenging to interpret and compare directly. To address this issue, we propose the following approach: for each model, we calculate the minimum and maximum possible risk values based on patients with the most favorable and least favorable characteristics at the time of transplantation. We then categorize the predicted risk scores for each of the three patients within these defined ranges. By positioning each patient within the risk spectrum of each model, we can classify them into low, medium, or high-risk levels. This process is visually illustrated in Figure 2.

After implementing this procedure, where each model determines the minimum and maximum risk values by testing all possible combinations of parameters from our dataset, and subsequently positioning the initial predictions for our test patients within these ranges, we can draw several conclusions. These individualized risk assessments provide clinicians with a data-driven tool to evaluate the potential benefits and risks of proceeding with aHSCT, facilitating more informed decision-making tailored to each patient’s unique clinical profile. These results are consistent with the criteria set by the physicians and the actual outcomes observed for these patients.

The models corroborate the lower risk expectation for Patients 1 and 2, who were anticipated to have a lower risk post-transplant. Both patients remain alive for 47 and 26 months following the procedure, respectively. The transplant has benefited these individuals, leading to improved survival outcomes as anticipated.

In the case of Patient 3, who passed away a few years after the operation, the models suggest that the intervention was still advisable, albeit with additional precautions. Although there was an associated risk, the procedure presented a reasonable chance of success, contingent upon careful monitoring and potential post-operative interventions.

Conversely, for the last patient, who exhibited more unfavorable characteristics at the time of transplantation and had a history of relapse following previous transplants, the outcome was markedly different. This patient succumbed within a year after the procedure. The models predicted that proceeding with the transplant would not be advisable in this scenario, as the likelihood of obtaining significant benefits from the treatment was minimal.

Critical ethical and medical considerations are raised when the predicted survival time with the operation does not substantially differ from the expected time without intervention. In such instances, the burden of undergoing an intensive procedure like aHSCT, combined with the associated risks and costs, may outweigh the potential benefits, making a non-interventional approach a more compassionate and medically sound decision.

The integration of these risk stratification results into clinical workflows can significantly enhance the decision-making process. By embedding risk scores into clinical practice, physicians could receive automated risk assessments alongside standard patient data, enabling real-time evaluation during multidisciplinary transplant board meetings. These risk predictions can aid in patient selection, inform treatment planning, and help prioritize post-transplant monitoring strategies based on individual risk profiles. In high-stakes procedures like aHSCT, where the balance between potential benefit and risk is critical, the use of predictive risk scores can contribute to more personalized, transparent, and ethically sound clinical care.

## 5 Conclusions and future work

This study has demonstrated the significant potential of *Survival Analysi*s (SA) models in predicting patient outcomes following *autologous Hematopoietic Stem Cell Transplantation* (aHSCT). By concentrating on risk predictions, we provide clinicians with actionable insights into the likelihood of adverse outcomes based on each patient’s pre-transplant clinical profile. This capability enables the early identification of individuals who may require closer monitoring and tailored post-transplant interventions, enhancing the overall approach to patient care.

SA models can handle censored data, a common challenge in medical research. Unlike traditional *Machine Learning* (ML) models, which often struggle with incomplete event data, SA models excel by delivering accurate risk predictions, even when not all patients have experienced the event of interest during the observation period. These risk scores allow clinicians to categorize patients into distinct risk levels, ranging from low to high, facilitating a more targeted approach to patient management.

A key finding of this study is that, while all evaluated models produced strong *Concordance Index* (C-index) results, further analysis using feature importance techniques revealed essential distinctions. Some models, such as Coxnet and Random Survival Forest, employed a broader array of clinical attributes, effectively capturing more available information to differentiate between patients. In contrast, despite their overall strong performance, gradient-boosting models often relied on a narrower subset of attributes, limiting their ability to distinguish between patients with similar characteristics. This underscores the importance of examining how models interpret clinical data to ensure comprehensive and reliable risk predictions.

Moreover, we could interpret the predicted risk scores in a clinically meaningful context by applying these models to test patients. The risk scores were translated into percentage-based rankings, enabling us to assess the appropriateness of the transplant for each patient. The transplant proved beneficial for those classified with low to moderate risk, as they survived several years post-procedure. However, for higher-risk patients, especially those with prior relapses or more severe conditions, the models indicated that the transplant was less likely to confer long-term survival benefits. In one instance, the models predicted that the procedure would likely not significantly enhance the patient’s outcome, reinforcing the need for careful evaluation in cases where the operation’s risks may outweigh its potential advantages.

Future research will focus on extending these models to predict time-to-event outcomes. Specifically, we aim to develop models that estimate how long a patient might expect to live following a transplant based on their pre-transplant characteristics. These time-to-event predictions could provide valuable insights for both clinicians and patients, allowing them to make more informed decisions regarding treatment options. Such predictions will be beneficial in situations where the decision to proceed with a transplant is uncertain, as they will offer more precise guidance on whether the procedure is likely to extend the patient’s life meaningfully or if alternative treatments should be considered.

In conclusion, this study emphasizes the importance of integrating advanced *Artificial Intelligence* (AI) and ML techniques, particularly SA models, into clinical practice for predicting patient risks after aHSCT. As we continue refining these models, their capacity to predict risk and survival time will enhance personalized treatment strategies, leading to improved patient care and better survival outcomes.
